# Kolumbo submarine volcano (Greece): An active window into the Aegean subduction system

**DOI:** 10.1038/srep28013

**Published:** 2016-06-17

**Authors:** Andrea Luca Rizzo, Antonio Caracausi, Valèrie Chavagnac, Paraskevi Nomikou, Paraskevi N. Polymenakou, Manolis Mandalakis, Georgios Kotoulas, Antonios Magoulas, Alain Castillo, Danai Lampridou

**Affiliations:** 1 Istituto Nazionale di Geofisica e Vulcanologia, Sezione di Palermo, Italy; 2 CNRS, Géosciences Environnement Toulouse, 14 Avenue Edouard Belin, Toulouse, France; 3 Department of Geology and Geoenvironment, National and Kapodistrian University of Athens, Panepistimiopolis, Zographou, Greece; 4 Hellenic Centre for Marine Research, Institute of Marine Biology, Biotechnology and Aquaculture, Heraklion Crete, Greece

## Abstract

Submarine volcanism represents ~80% of the volcanic activity on Earth and is an important source of mantle-derived gases. These gases are of basic importance for the comprehension of mantle characteristics in areas where subaerial volcanism is missing or strongly modified by the presence of crustal/atmospheric components. Though, the study of submarine volcanism remains a challenge due to their hazardousness and sea-depth. Here, we report ^3^He/^4^He measurements in CO_2_–dominated gases discharged at 500 m below sea level from the high-temperature (~220 °C) hydrothermal system of the Kolumbo submarine volcano (Greece), located 7 km northeast off Santorini Island in the central part of the Hellenic Volcanic Arc (HVA). We highlight that the mantle below Kolumbo and Santorini has a ^3^He/^4^He signature of at least 7.0 Ra (being Ra the ^3^He/^4^He ratio of atmospheric He equal to 1.39×10^−6^), 3 Ra units higher than actually known for gases-rocks from Santorini. This ratio is also the highest measured across the HVA and is indicative of the direct degassing of a Mid-Ocean-Ridge-Basalts (MORB)-like mantle through lithospheric faults. We finally highlight that the degassing of high-temperature fluids with a MORB-like ^3^He/^4^He ratio corroborates a vigorous outgassing of mantle-derived volatiles with potential hazard at the Kolumbo submarine volcano.

Most of the volcanic activity worldwide occurs in the oceans^1^, with newly formed volcanoes at mid-ocean ridges, hot spots and volcanic island arcs. Any submarine volcanic eruption leads to a major regional disruption of the environment by modifying the chemical composition of adjacent seawaters and releasing a large amount of gases with consequences on the associated deep-sea ecosystems and atmospheric chemistry^2^,^3^,^4^. This degassing can be even more catastrophic when high volumes of volcanic gases reach the atmosphere and become lethal for humans, as occurred in 1650 A.D. at Kolumbo volcano (South Aegean Sea; Fig. 1a,b). During this submarine eruption ~70 people were killed at the nearby island of Santorini^5^. Thus, the study of submarine volcanic degassing is of great importance to better constrain the characteristics and evolution of geodynamic settings showing elevated volcanic hazard with considerable impact on the daily life of local populations.

The geochemical studies carried out on submarine fluids have been mainly focused on mid-ocean ridge hydrothermal systems^6^,^7^,^8^, whereas only a few of them investigated subduction zone environments^9^,^10^,^11^,^12^. The HVA in the South Aegean Sea (East Mediterranean) results from the subduction of the African plate below the European one^13^,^14^ (Fig. 1a). In the last decades, several studies were dedicated to better constrain the mantle characteristics below the HVA and understand the dynamics of this complex geodynamic setting but a general understanding has not been reached thus far^15^,^16^,^17^,^18^,^19^,^20^,^21^,^22^. The recent discovery of the high-temperature hydrothermal field at the Kolumbo submarine volcano^23^ (Fig. 2a–c), located 7 km northeast off Santorini Island (Fig. 1b), has opened up a new opportunity to deepen our knowledge of Aegean mantle features. Indeed, Kolumbo is considered to be the most active volcanic system of the region at present time^15^,^23^,^24^,^25^,^26^. Its magmatic activity is evidenced by numerous hydrothermal sites venting vigorously and continuously up to ~220 °C warm fluids^27^ and CO_2_-rich gases at the crater seafloor (Fig. 2a–c)^4^. This ongoing intense degassing contrasts radically with the low-temperature phenomena typically observed in the Santorini caldera, next to the Kameni islands^4^,^23^,^28^ (Fig. 1b).

The Santorini-Amorgos Basin surrounding Kolumbo system, divides the HVA into a seismic-active eastern zone and a relatively quiet western area^29^. The main seismic hypocentres beneath Kolumbo are located at depths of 6–9 km^24^, indicating a high level of magmatic activity below this volcano. Accordingly, this range of depth corresponds to the location of the main magma chamber^24^,^25^ (Fig. 3).

The Kolumbo hydrothermal system is poorly studied^4^,^30^, and little is known about the origin of fluids escaping from its seafloor (Fig. 2c) and the potential link with those emitted from Santorini subaerial vents, as well as other active volcanoes across the HVA. Here, we report on the ^3^He/^4^He ratios of gases discharging from seven different hydrothermal chimneys of the Kolumbo submarine volcano in order to constrain the He isotopic characteristics of the mantle beneath this key sector of the HVA. We focus on the ^3^He/^4^He ratio because this is the most powerful geochemical tracers to define the origin of volatiles released from the solid earth and the magmatic/mantle features^31^,^32^. Indeed, ^3^He has a primordial origin and is preferentially degassed from the Earth’s interior. Because He is highly mobile, chemically inert, physically stable and nonbiogenic, the interaction of this noble gas on its movement toward the surface is minimized, and its isotopic composition is not affected by subsequent chemical reactions^8^. It also gives unambiguous signals of magma rising up in volcanic plumbing systems^33^,^34^,^35^,^36^.

## Geodynamic setting

**Figure 1:**
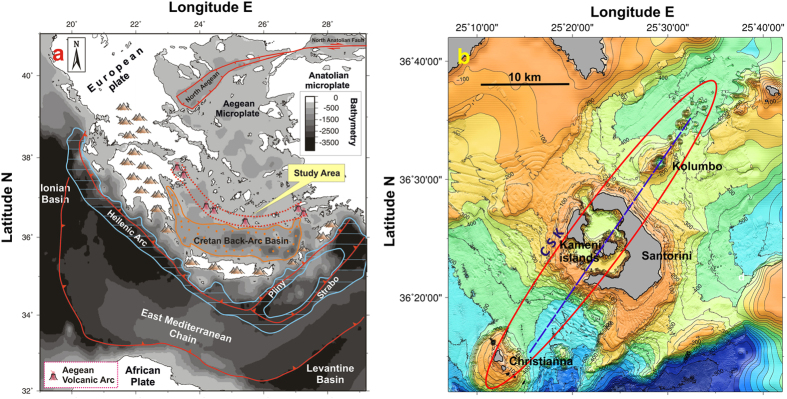
(**a**) Simplified map of the present day geodynamic structure of the HVA, showing the modern volcanic arc developed behind the Hellenic trench, the Peloponnese–Crete island arc and the Cretan back-arc basin. The study area is located in the center of the HVA (modified from ref. 28). (**b**) Swath bathymetric map of Christianna-Santorini-Kolumbo (CSK) volcanic fields (modified from ref. 26). The location of the NE-SW profile of Fig. 3 is also reported (purple dotted line), as well as CSK tectonic alignment.

The HVA is a 500 km-long curving chain of volcanoes, Pliocene to modern in age, extending from the Methana peninsular zone on the Greek mainland, through the islands of Milos-Antimilos, Santorini, Kos-Nisyros, towards the western peninsular zone in Turkey. The HVA results from the extended and elongated subduction of the African plate beneath the Eurasian continent (Fig. 1a). The plate horizontal velocities measured by GPS in the Eastern Mediterranean highlight the presence of a clockwise rotation of a broad region relative to Eurasia at rates in the range of 20–30 mm/yr^19^,^22^. This rotation is combined with the continuing southward rollback of the Hellenic subduction around a pole located in Albania^19^,^22^. The complex regime of movements causes a NW–SE extension in the Western Anatolia–Aegean system, which in turn leads to the thinning of the Aegean crust from roughly 50 to 25 km^19^. Based on seismic tomography models, the Aegean subduction is characterized by a single slab, more than 1,500 km-long, which extends down to the lower mantle^22^. These models also suggest that the slab does not extend far eastward and that possible ruptures are present below Nisyros volcanic system and western Turkey, where modern alkaline magmatism is present^15^,^22^. In addition, P-wave velocities along the HVA indicate the presence of a low velocity zone just above the downgoing slab, which can be interpreted as the occurrence of asthenospheric mantle^18^,^37^. As a result, the interface between the slab and mantle wedge is influenced by primary magmas generated in the underlying asthenosphere^18^.

Within the HVA, Santorini volcano is one of the most active and it is very famous worldwide because of its large explosive eruption that occurred in Bronze Age, which caused significant impacts to human populations in the eastern Mediterranean^38^. In the Santorini area, volcanism extends northeastward into the submarine environment evidenced by a series of small craters and cones aligned along Christianna-Santorini-Kolumbo (CSK) tectonic line^28^, which likely controls the pathways of hydrothermal circulation within the region (Fig. 1b). This is a vertical northeast trending zone, up to 30–40 km large and 45 km deep^29^. The CSK line created an area of structural weakness, characterized by increased seismic activity^25^, enabling the upward migration of fluids at Kolumbo. The focus of this study is dedicated to a better understanding of the composition of these fluids in order to further constrain the mantle features within the HVA.

## Results

**Figure 2:**
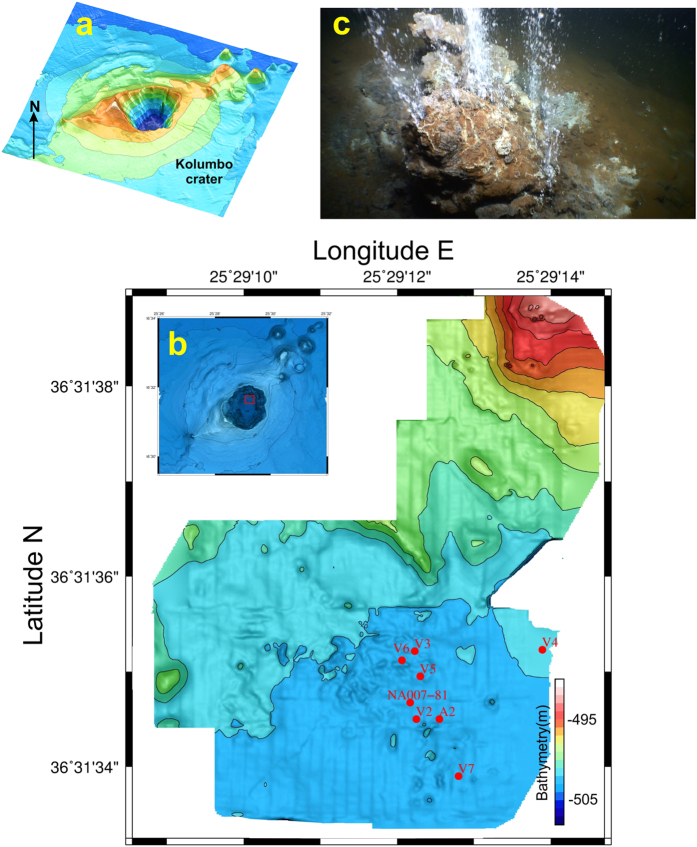
(**a**) 3D Bathymetric map of Kolumbo submarine volcano, showing the shape of the crater in whose bottom are located the hydrothermal chimneys; (**b**) High resolution swath bathymetric map of Kolumbo crater bottom (Fig. 1c) with the location of the sampled hydrothermal chimneys as labeled in Table 1. (**c**) Active hydrothermal vent discharging both gases (>99% CO_2_) and high-temperature fluids collected in this work.

The chemical composition of gases collected from each of the seven chimneys (Fig. 2b,c) consists of almost pure CO_2_ (Table 1), in line with the findings of previous work at the same area^4^. This feature is typical of submarine volcanic emissions and it has also been observed either in hot spot systems as Loihi^39^ (Hawaii), or in subduction-related environments like Panarea (Aeolian islands, Italy^9^), offshore NE Taiwan^40^, along the Mariana and Tonga-Kermadec arcs^11^. The O_2_ (<2.1%) and N_2_ (<8.5%) concentration measured in Kolumbo gases suggests a contamination by atmospheric air, which, nevertheless, has a minor influence on helium isotopic composition, as evidenced by the ^4^He/^20^Ne ratio (9–270; Table 1) being 30 to 250 times higher than that of ambient air (^4^He/^20^Ne = 0.318). At the bottom of Kolumbo crater, seawater exhibits acidic conditions (pH = 5), as a consequence of CO_2_ release and dissolution^3^,^4^,^30^. This dissolution, the extent of which varies depending on the actual flux of gas coming out from each vent, is responsible for the variable helium concentrations (15–41 part per million) measured in our samples (Table 1).

The ^3^He/^4^He ratios of seven gas samples are virtually identical to one another, irrespective of the vent being sampled and the extent of CO_2_ dissolution, and vary in the narrow range of 7.0–7.1 Ra (Fig. 4a and Table 1). These values are slightly higher than the single previous measurement of 6.8 Ra reported for Kolumbo^4^. Because the ^3^He/^4^He ratio does not suffer any significant fractionation during gas-water interaction, it can be used to assess the origin of emitted fluids.

## Discussion

### Kolumbo-Santorini volcanic system

The ^3^He/^4^He ratios measured at Kolumbo (7.0–7.1 Ra) are within the typical range of values found in arc volcanoes worldwide and correspond to a MORB-like ^3^He/^4^He signature of 8 ± 1 Ra^8^,^41^ (Fig. 4a). This finding indicates that magma degassing beneath Kolumbo and feeding its hydrothermal system has a ^3^He/^4^He representative of the primary source. This signature is surprisingly higher (more than 3 Ra units) than that measured in gases and rocks (fluid inclusions) of the adjacent (<7 km) Santorini island^34^ (Fig. 4a). Indeed, gases collected between 1988 and 2001 from both Nea and Palea Kameni islets were in the range of 3.2–3.8 Ra^17^,^42^,^43^,^44^, while during the 2011–2012 seismic, geodetic and geochemical unrest these ratios increased from 3.6 to 4.0 Ra (ΔRa = 0.4)^34^. Also, the ^3^He/^4^He ratios measured in some mafic enclaves, hosted by dacitic lavas recently erupted at Nea Kameni, yielded a range of 3.1–3.6 Ra^34^ (Fig. 4a). All these data imply that the magma residing at shallow depths into the Santorini plumbing system has a distinct and fairly consistent He-isotope signature ranging between 3 and 4 Ra.

Substantial spatial variations of the ^3^He/^4^He ratios over a 10-km distance have been recently observed at the mid oceanic ridge (7.5–10.2 Ra^45^). However, to the best of our knowledge, it is rare that two distinct and active volcanic systems located only a few km apart from one another display at the same time such a wide variation in ^3^He/^4^He ratio. This finding emphasizes the importance of investigating fluids being discharged by submarine volcanic clusters with sufficient spatial coverage and resolution, if precious insights about the mantle are to be gained around the planet. In light of this, while the high ^3^He/^4^He ratios at Kolumbo can be considered representative of the mantle source beneath the submarine volcano, the lower ^3^He/^4^He values measured at Santorini lead to two fundamental questions:

(1) Do the ^3^He/^4^He ratios measured at Santorini reflect a local mantle heterogeneity, despite the very short distance among the two volcanic systems?

(2) Do these ratios reflect an addition by radiogenic ^4^He within the Santorini plumbing system of magma and/or fluids originated from a homogeneous mantle?

To decipher between the two hypotheses, we compare the lowest ^87^Sr/^86^Sr ratios measured in whole rocks from Santorini (0.7035^15^,^46^,^47^) with ^3^He/^4^He in gases and fluid inclusions of the two volcanic systems (Fig. 4b). For completeness, in Fig. 4b we report the range of ^87^Sr/^86^Sr ratios measured in bulk rocks erupted in each volcanic system. In magmatic environments, these geochemical tracers are inversely correlated, in the sense that the highest He isotopic ratios correspond to the lowest Sr isotopic ratios, which are measured in the most mafic and primitive products, and vice versa. This behaviour has been observed in other magmatic systems worldwide^48^,^49^, and is simplified in the insert diagram of Fig. 4b in which we plot a binary mixing line between a MORB-like mantle (^3^He/^4^He = 8.0 Ra; ^87^Sr/^86^Sr = 0.7020) and a hypothetical Aegean continental crust (^3^He/^4^He = 0.03 Ra; ^87^Sr/^86^Sr = 0.7150)^50^,^51^. We obtain a straight line because for simplicity we assumed *k* = 1 (being *k* = (He/Sr)_crust_/(He/Sr)_mantle_), however slight variations of this ratio do not modify our inferences. Based on this mixing, the right y axis of plot 4b reports the expected ^3^He/^4^He ratios corresponding to the ^87^Sr/^86^Sr ratios reported in the left y axis.

**Figure 3:**
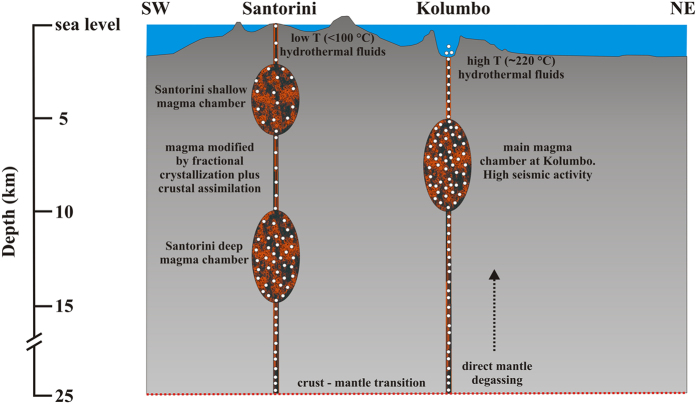
Cross section along ~40 km NE–SW profile (reported in Fig. 1b) moving from the Kolumbo area towards Santorini Island showing a sketch of the distinct plumbing systems beneath the two volcanoes. The depth of the magmatic chambers (dimensions not to scale) and the main volcanological, seismic and petrologic features characterizing Santorini and Kolumbo volcanic systems are also reported^15^,^23^,^24^,^25^,^28^,^29^,^55^,^57^.

**Figure 4:**
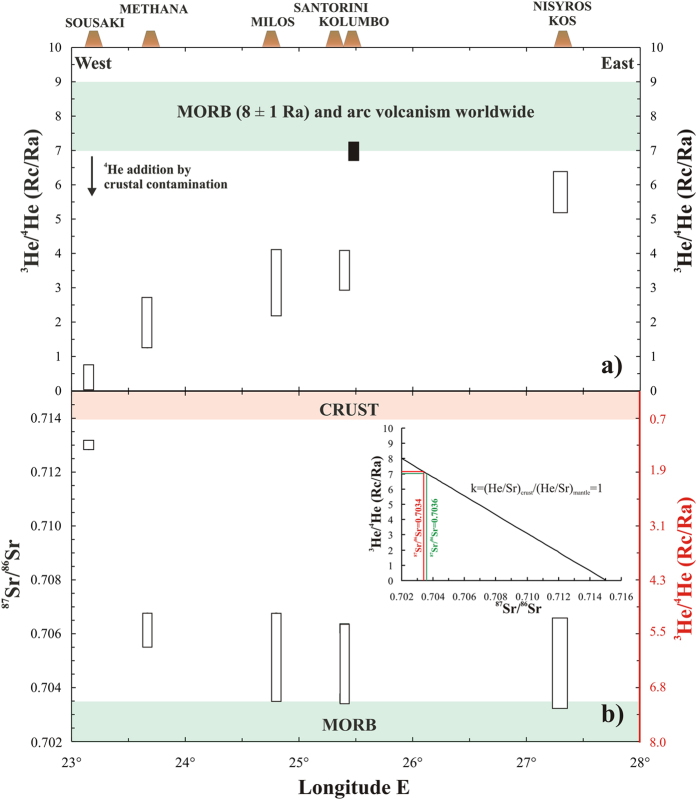
Along-arc variations of (**a**) ^3^He/^4^He^4^,^17^,^34^,^42^,^43^,^44^ and (**b**) ^87^Sr/^86^Sr ratios in the HVA^15^,^16^,^46^,^47^. Solid black rectangle in plot 4a reproduces the range of He isotope ratios of Kolumbo gases. In the insert diagram of plot 4b we report a binary mixing between a MORB-like mantle (^3^He/^4^He = 8.0 Ra; ^87^Sr/^86^Sr = 0.7020) and a hypothetical Aegean continental crust (^3^He/^4^He = 0.03 Ra; ^87^Sr/^86^Sr = 0.7150)^50^,^51^. See text for further details. Based on this mixing, the secondary y axis of plot 4b reports the expected ^3^He/^4^He ratios (red coloured) corresponding to the ^87^Sr/^86^Sr ratios reported in the left y axis.

Petrological studies on the HVA indicated that volcanic rocks from Santorini display compositional features typical of subduction-related tectonic settings, with a high variability of trace elements and Sr-Nd-Pb isotopes mainly ascribed to crustal assimilation processes^15^,^46^,^47^. Considering that the most primitive products erupted at Santorini showed ^87^Sr/^86^Sr values as low as 0.7035^15^,^46^,^47^ (Fig. 4b), the genesis of parental magmas can be ascribed to the partial melting of a depleted, MORB-like, mantle wedge^15^,^18^,^47^,^52^. As shown in the insert plot of Fig. 4b, these ^87^Sr/^86^Sr values are highly consistent with the ^3^He/^4^He ratios measured in gases at Kolumbo (7.0–7.1 Ra; see also secondary y axis in Fig. 4b). Conversely, the ^3^He/^4^He ratios measured in gases and rocks of Santorini (3.1–4.0 Ra) cannot account neither for the very low ^87^Sr/^86^Sr measured in basalts, nor for the ratios measured in mafic enclaves hosted in dacitic lavas of Nea Kameni (^87^Sr/^86^Sr~0.7048^34^). This implies that the wide variability of Sr isotope composition observed among the erupted volcanic at Santorini (^87^Sr/^86^Sr from 0.7035 to 0.7062; Fig. 4b) is related to magmatic differentiation which predominantly takes place in a shallow magma chamber plus assimilation of crustal rocks during magma ascent plays an important role in the evolution of Santorini magmas^15^,^46^,^47^ (Fig. 3). Accordingly, several models of assimilation plus fractional crystallization involving Santorini basalts as mafic end-members and crustal basement rocks as contaminants have been proposed^18^,^46^,^47^.

The composition of rocks erupted from Kolumbo is much less investigated than in Santorini. Available data indicated that rocks erupted from Kolumbo volcano range from andesites to rhyolites and belong to calcalkaline series, similarly to most of the Santorini rocks^15^. Pumices are the main products of the last Kolumbo eruption at 1650 A.D. and lie along a magmatic evolution line similar to the suite of silicic pyroclastics from the nearby Santorinivolcano^53^. Unfortunately, no Sr isotopic data have been published yet for Kolumbo rocks.

Recent volcanological and geochemical investigations suggested the presence of two distinct plumbing systems beneath Santorini and Kolumbo volcanic systems^15^,^24^ (Fig. 3). These two reservoirs are also characterized by distinct geochemical and mineralogical characteristics, suggesting that primary magmas beneath Santorini are modified by successive processes of crustal assimilation plus fractional crystallization and magma mixing^15^,^18^,^24^. As already highlighted^34^, the occurrence of magma contamination by crustal rocks in Santorini plumbing system would eventually lead to the reduction of ^3^He/^4^He ratio with regard to parental magmas. On the other hand, magmas feeding Kolumbo would derive directly from the mantle^24^.

Santorini and Kolumbo volcanic systems lie along the same tectonic alignment that starts from Christianna north-eastwards (CSK^28^, Fig. 1b) and share a common region of lithospheric weakness. This unequivocally indicates that these volcanic systems originated from the same geological structure, but distinct magmatic reservoirs are active in the crust beneath each one of them^15^,^24^ (Fig. 3). Furthermore, our data argue that the mantle below Kolumbo and Santorini can be considered homogeneous in terms of He isotope ratios (^3^He/^4^He ≥ 7.0 Ra). The observed He signature is MORB-like, as in most arc volcanoes worldwide in which the subduction of oceanic crust does not affect He isotopic composition of the mantle wedge^8^,^41^.

If petrological features argue for primary magma undergoing differentiation plus crustal assimilation process in the magmatic plumbing systems below Santorini and Kolumbo volcanoes, then why do we observe ^3^He/^4^He ratios as high as 7.1 Ra only at Kolumbo? Two possible hypotheses can be proposed:

(1) Evolution and crustal assimilation by magma beneath Kolumbo are very low and do not imply a contribution of ^4^He-rich rocks and/or fluids;

(2) Predominant degassing at Kolumbo occurs directly from the mantle and fluxes the plumbing system, overprinting the He isotopic signature of evolved/contaminated magmatic products.

The geological features following a cross section through Santorini and Kolumbo volcanoes indicate a similar crustal thickness (~25 km^54^; Fig. 3) and common basement rocks^24^,^30^, discarding the first hypothesis. Furthermore, local seismological studies carried out in the area revealed an active tectonic regime beneath Kolumbo volcano characterized by the presence of faults^24^,^55^, whereas there is no evidence of continuous seismic activity beneath the caldera of Santorini volcano with the exception of unrest periods^24^,^25^,^29^,^56^. These geological and geophysical observations are in very good agreement with recent volcanological and marine studies that showed an intense high-temperature hydrothermal activity at Kolumbo, in comparison to the low-level activity of the Santorini caldera (submarine and subaerial fluid temperatures <100°)^23^,^28^,^34^. As a result, we conclude that magma residing in the shallow plumbing system beneath Santorini could be volumetrically small, strongly degassed in terms of helium, and prone to the crustal-derived ^4^He contamination by surrounding rocks. On the other hand, the ^3^He/^4^He ratios at Kolumbo could be the result of mantle degassing (fluxing) in a regional extensive regime characterized by the presence of active lithospheric faults beneath the central part of the HVA (Fig. 1b), which would overprint the signature of ^3^He/^4^He of evolved/contaminated magma residing at shallow depths, thus allowing to measure mantle ratios (Figs. 3 and 4a).

Under this prism, the Kolumbo submarine volcano is conceived to represent a sort of “window” into the mantle of the central HVA.

### Geodynamic implications on the Hellenic Volcanic Arc

The ^3^He/^4^He ratios of Kolumbo (up to 7.1 Ra) are also the highest ever measured in gases and rocks belonging to the HVA (Fig. 4a). Until now, the highest values were measured in gases emitted at Nisyros (6.2 Ra), located at the eastern side of the arc^17^. In this case, the presence of crustal assimilation at shallow depths below the island or the addition of radiogenic ^4^He to a MORB-like mantle wedge were considered unlikely, while the ^3^He/^4^He ratio of Nisyros was deemed to be representative of South Aegean mantle and consistent with the European Sub-Continental Lithospheric Mantle (SCLM; 6.1 ± 0.9 Ra). The same study identified a regional trend toward a westward decrease of ^3^He/^4^He ratios from Nisyros to Sousaki volcanoes (Fig. 4a) and it was attributed to a variable degree of crustal assimilation and/or to different magmatic activity in each volcanic system^17^.

Our current ^3^He/^4^He measurements from Kolumbo provide new constrains on these interpretations, because the mantle below the HVA (at least in its central sector, i.e., Kolumbo-Santorini) has an He isotopic signature compatible with a MORB-like source, and the high ^3^He/^4^He ratios at Kolumbo rule out the presence of a regional trend. In order to evaluate if the mantle signature beneath Kolumbo may extend up to Nisyros, we used the same approach described in the previous section based on He and Sr isotope signatures in gases and rocks. Petrological studies of the HVA indicate that the most primitive products erupted at Nisyros are characterized by ^87^Sr/^86^Sr values as low as 0.7034^15^,^46^ (Fig. 4b). These Sr isotope ratios are perfectly compatible with the high ^3^He/^4^He ratios measured at Kolumbo (7.0–7.1 Ra; Fig. 4b), indicating that this He isotopic signature can be considered also representative of the mantle below Nisyros. On the other hand, ^3^He/^4^He ratios of 6.2 Ra would be compatible with ^87^Sr/^86^Sr of ~0.7049 (Fig. 4b). The ^87^Sr/^86^Sr variability of Nisyros (0.7034–0.7064^15^,^46^), which is in line with measured values at Santorini, shows that magmatic differentiation plus assimilation of crustal rocks during magma ascent could also occur in the evolution of Nisyros magmas. The occurrence of these processes in the plumbing system of the volcano is reasonably responsible of the slight lowering of ^3^He/^4^He ratios at Nisyros (6.2 Ra) compared to the pristine values obtained at Kolumbo volcano (7.0–7.1 Ra; Fig. 4a).

These new considerations can be extended westward up to Milos volcanic system, whose lowest ^87^Sr/^86^Sr (i.e., 0.7037^15^) are comparable to those measured at Santorini and Nisyros (Fig. 4b). On this ground, the ^3^He/^4^He ratios <4 Ra^17^,^44^ (Fig. 4a) in our opinion indicate strong crustal contamination process that would mask the pristine isotope signature. On the other hand, the westernmost part of the HVA (from Methana to Sousaki) showed the lowest ^3^He/^4^He (0.17–2.6 Ra^17^,^44^) and ^87^Sr/^86^Sr values >0.7055 even in the most mafic volcanics (basaltic-andesite to andesite of Poros and Methana^15^,^46^) (Fig. 4a,b). A recent investigation based on Sr and Nd isotopes highlighted an extensive crustal assimilation at Methana, higher than in Santorini and Nisyros^46^. These features strongly support the recognized variability of the He isotopic signature in these volcanic systems, even if magma mixing and mingling could simultaneously have occurred to generate the recognized variability in the petrological features of erupted volcanics^46^.

Based on these arguments, we propose that the mantle below the central and eastern part of the HVA (from Milos through Santorini-Kolumbo up to Nisyros-Kos) is rather homogeneous in terms of ^3^He/^4^He and preserves its MORB-like magmatic signature^8^,^41^.

### Volcanic hazard at Kolumbo

Among the subaerial volcanoes belonging to the HVA, Nisyros and Santorini volcanoes are considered as the most active ones. In 1995–1998, the former was characterized by a seismic activity, after which the geochemical monitoring of gases collected from the fumaroles highlighted an increase of the ^3^He/^4^He (∆Ra = 0.7)^17^. This variation was related to the upward movement of subsurface magma, which triggered an enhanced contribution of mantle helium^17^. In 2011–2012, a seismic, geodetic and geochemical unrest took place in Santorini^56^, during which an increase of ^3^He/^4^He ratios (∆Ra = 0.4) has been recorded in the fluids emitted at Nea and Palea Kameni^34^. This increase was interpreted as the result of the intrusion of a more-primitive ^3^He-rich magma into the shallow plumbing system. These unrests observed at Nisyros and Santorini led to a general fear that an eruption could be imminent.

Based on our present results, we infer that Kolumbo submarine volcano is very active at present time and the associated volcanic hazard may be potentially high. With this in mind, the development of a regular geochemical monitoring program for this potentially dangerous submarine volcano is strongly suggested.

In summary, hydrothermal fluids from Kolumbo submarine volcano exhibited the highest ^3^He/^4^He ratios (up to 7.1 Ra) across the HVA, which are surprisingly higher (>3 Ra units) than those previously reported for the adjacent island of Santorini. These fluids reflect a direct mantle degassing that can only be produced in an extensive tectonic regime characterized by the presence of active lithospheric faults. The range of ^3^He/^4^He ratio measured at Kolumbo is the highest of all the HVA and is representative of the mantle below the central and eastern part of the HVA. The degassing of high-temperature fluids from the bottom of Kolumbo crater with a mantle-like ^3^He/^4^He ratio suggests that this submarine volcano is characterized by a vigorous activity with potential volcanic hazard.

## Methods

### Sampling

The Kolumbo submarine volcano is characterized by a well-defined 1500-meter-wide crater (Fig. 2a), with a rim as shallow as 17 m and a floor ~500 m below the sea level^26^,^28^ (Fig. 2a,b). On the active hydrothermal vent field at the crater floor of Kolumbo (Fig. 2b), first discovered in 2006^23^, relatively high temperatures (up to ~220 °C^4^,^27^) have been measured. During the 4-SeaBiotech cruise on board of the *R/V Aegaeo* (Hellenic Centre for Marine Research) in May 2014, seafloor exploration of hydrothermal activity has been carried out with the Greek ROV Max Rover. Due to the pressure-temperature (P-T) conditions of the vents (P~50 bar, T > 200 °C), hydrothermal fluid/gas discharges are in form of clear waters together with continuous gas bubbling at different chimneys (Fig. 2c and supplementary movies).

High temperature hydrothermal gases were collected in titanium gas-tight bottles of 200 ml capacity each equipped with funnels. These bottles have been built specially to avoid out-gassing and gas leakage during recovery and were initially designed by IFREMER for the manned submersible Nautile. Additional samplers has been projected and developed by Istituto Nazionale di Geofisica e Vulcanologia (INGV), Sezione di Palermo (Italy), and experimentally used at Kolumbo. Both types of bottles have been pre-evacuated on board of the oceanographic vessel, just before each submersion (internal pressure <10^−3^ bar). We point out that we did not observe any difference in using the two types of gas-tight samplers. For sake of clarity, sample A2 was collected with INGV-type bottle (Table 1). The gas-tight bottles were held by the ROV Max Rover of HCMR arm over the bubble streams discharged from the chimneys and then triggered to collect a sample (see supplementary movies).

We collected gas samples from seven different chimneys located at the bottom of Kolumbo crater (Fig. 2b,c). After each sampling, once the gas-tight syringes were onboard of the research vessel, gases at 50 bar were immediately extracted in stainless steel or titanium bottles for safe transportation (at lower pressures) towards the laboratory.

### Analytical techniques

The analysis of chemical composition and He-Ne isotopes of the collected gases has been performed in the laboratories of INGV-Palermo. The concentrations of CO_2_, O_2_ and N_2_ were analyzed by a Perkin Elmer Clarus 500 gas chromatograph equipped with a 3.5-m Carboxen 1000 column and double detector (hot-wire detector and flame ionization detector), with analytical errors of <3%. ^3^He, ^4^He and ^20^Ne and the ^4^He/^20^Ne ratios were determined by separately admitting He and Ne into a split flight tube mass spectrometer (GVI-Helix SFT, for He analysis) and into a multicollector mass spectrometer (Thermo-Helix MC plus, for Ne analysis), after standard purification procedures^32^,^34^. The ^3^He/^4^He ratio is expressed as R/Ra (being Ra the He isotope ratio of air and equal to 1.39·10^−6^)^58^, and the analytical error is generally below 0.3%. The ^3^He/^4^He values were corrected for the atmospheric contamination based on the measured ^4^He/^20^Ne ratio^34^ as follows:


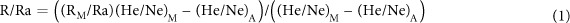


where subscripts M and A refer to measured and atmosphere theoretical values, respectively [(He/Ne)_A_ = 0.318]^58^. The corrected ^3^He/^4^He ratios reported in the text and in Table 1 are expressed as Rc/Ra values. The correction is small or negligible for most of the samples, with the maximum bias of ~0.2 Ra appearing in the sample showing the lowest ^4^He/^20^Ne.

## Additional Information

**How to cite this article**: Rizzo, A. L. *et al.* Kolumbo submarine volcano (Greece): An active window into the Aegean subduction system. *Sci. Rep.* **6**, 28013; doi: 10.1038/srep28013 (2016).

## Supplementary Material

Supplementary Information

Supplementary Movie 1

Supplementary Movie 2

## Figures and Tables

**Table 1:** Analyses of major gaseous components, helium and neon in gases from Kolumbo hydrothermal vents.

Sample	Depth (m)	Latitude	Longitude	^4^He (ppm)	^20^Ne (ppm)	O_2_ (%)	N_2_ (%)	CO_2_ (%)	R/Ra	^4^He/^20^Ne	Rc/Ra	Error (+/−)
A2	497	36°31.5700′N	25°29.2110′E	23	0.09	0.17	1.1	98.0	7.01	270.3	7.02	0.13
V2	498	36°31.5700′N	25°29.2050′E	15	1.69	2.09	8.5	88.3	6.84	9.1	7.05	0.017
V3	498	36°31.5843′N	25°29.2046′E	26	0.10	0.10	0.9	98.4	7.04	248.3	7.05	0.011
V4	498	36°31.5846′N	25°29.2378′E	22	0.84	1.08	5.0	91.5	6.95	29.1	7.05	0.012
V5	500	36°31.5790′N	25°29.2060′E	25	0.51	0.67	3.1	95.4	7.05	48.0	7.10	0.016
V6	498	36°31.5824′N	25°29.2012′E	19	0.08	0.00	0.6	97.4	7.00	234.4	7.01	0.018
V7	498	36°31.5580′N	25°29.2160′E	41	0.29	0.17	1.3	96.7	6.98	139.4	7.00	0.015
NA007-081*	502	36°31.5735′N	25°29.2034′E	24	0.03	0.01	0.3	99.4	6.84	990.0	6.84	–

^*^Sample from ref. 4.
