# Supplementary material for: Kolumbo submarine volcano (Greece): An active window into the Aegean subduction system
Source: Sci Rep. 2016 Jun 17;6:28013. doi: 10.1038/srep28013 (PMC4911562; doi:10.1038/srep28013)
Supplement: Supplementary Information [file srep28013-s1.pdf]

Kolumbo submarine volcano (Greece): An active window into the Aegean subduction system

Andrea Luca Rizzo[1]*, Antonio Caracausi[1], Valèrie Chavagnac[2], Paraskevi Nomikou[3], Paraskevi N. Polymenakou[4], Manolis Mandalakis[4], Georgios Kotoulas[4], Antonios Magoulas[4], Alain Castillo[2], Danai Lampridou[3]

1 Istituto Nazionale di Geofisica e Vulcanologia, Sezione di Palermo, Italy

2 CNRS, Géosciences Environnement Toulouse, 14 Avenue Edouard Belin,Toulouse, France

3 Department of Geology and Geoenvironment, National and Kapodistrian University of Athens, Panepistimiopolis, Zographou, Greece

4 Hellenic Centre for Marine Research, Institute of Marine Biology, Biotechnology and Aquaculture, Heraklion Crete, Greece

*Corresponding author: Andrea Luca Rizzo, Istituto Nazionale di Geofisica e Vulcanologia, Via Ugo La Malfa 153, 90146 Palermo (Italy). Phone: (+39) 091 6809407, email: andrea.rizzo@ingv.it

Supplementary material

Supplementary Movie 1. Gas sampling with IFREMER gas tight sampler at one of the chimneys.

Supplementary Movie 2. Gas sampling with INGV-Palermo gas tight sampler at a strongly degassing chimney.
